# O.4.2-8 Gait differences between adults with COPD and healthy controls: systematic review and metaanalyses

**DOI:** 10.1093/eurpub/ckad133.183

**Published:** 2023-09-11

**Authors:** Joren Buekers, Laura Delgado Ortiz, Dimitrios Megaritis, Ashley Polhemus, Nikolaos Chynkiamis, Sara Buttery, Sofie Breuls, Sarah Koch, Elena Gimeno Santos, Heleen Demeyer, Nick Hopkinson, Ioannis Vogiatzis, Thierry Troosters, Anja Frei, Judith Garcia-Aymerich

**Affiliations:** ISGlobal, Barcelona, Spain; ISGlobal, Barcelona, Spain; Northumbria University Newcastle, Newcastle Upon Tyne, UK; University of Zurich, Epidemiology, Biostatistics and Prevention Institute, Zurich, Switzerland; Thorax Research Foundation, Athens, Greece; Imperial College, London, UK; Department of Rehabilitation Sciences, KU Leuven, Leuven, Belgium; joren.buekers@isglobal.org; ISGlobal, Barcelona, Spain; ISGlobal, Barcelona, Spain; Department of Rehabilitation Sciences, KU Leuven, Leuven, Belgium; joren.buekers@isglobal.org; Imperial College, London, UK; Northumbria University Newcastle, Newcastle Upon Tyne, UK; Department of Rehabilitation Sciences, KU Leuven, Leuven, Belgium; joren.buekers@isglobal.org; University of Zurich, Epidemiology, Biostatistics and Prevention Institute, Zurich, Switzerland; ISGlobal, Barcelona, Spain

## Abstract

**Purpose:**

Fall risk is increased in adults with COPD. Although gait is an important risk factor for falls, hospitalisations, and mortality, the available literature shows inconsistencies on whether gait differs between adults with COPD and healthy controls. The aim of this study was to identify differences in digitally-measured gait characteristics during laboratory tests between adults with COPD and healthy controls.

**Methods:**

As part of the Mobilise-D project (https://www.mobilise-d.eu), we conducted a systematic review built upon a previous scoping review (Polhemus, A. et al. npj Digit. Med. 2021). The search was updated in July 2021. Teams of two reviewers independently screened articles and extracted data. Meta-analyses were performed in studies not considered at a high risk of bias.

**Results:**

Twenty-five studies were included. Gait speed was studied in 17 studies (68%), step length in 9 (36%), step time in 7 (28%), cadence in 6 (24%) and step width in 5 (20%). Meta-analyses revealed that adults with COPD walked with a lower self-selected speed and top speed than healthy controls. There was a trend towards a lower step length, lower cadence and higher step time at a self-selected walking speed in adults with COPD compared to healthy controls. Step width was similar for both groups.

**Conclusion:**

Adults with COPD exhibit slower gait than healthy controls. This could contribute to the increased fall risk in adults with COPD.

**Support/Funding Source:**

This work was supported by the Mobilise-D project that has received funding from the Innovative Medicines Initiative 2 Joint Undertaking (JU) under grant agreement No. 820820.

